# Heightened effective connectivity of DLPFC-mPFC and DLPFC-ACC circuits in major depressive disorder with suicidal ideation: evidence from a TMS-EEG study

**DOI:** 10.1038/s41398-025-03515-z

**Published:** 2025-08-30

**Authors:** Meng Chen, Xingxing Li, Wenhao Zhuang, Yongming Xu, Zhenglei Pei, Jimeng Liu, Yuanyuan Zhang, Chang Yu, Yubo Wang, Xiaoli Liu, Junfang Zhang, Guangwei Hou, Yinping Chen, Miaomiao Xu, Yafang Tang, Yan Ding, Jie Zhang, Dongsheng Zhou

**Affiliations:** 1 Department of Psychology, Affiliated Kangning Hospital of Ningbo University (Ningbo Kangning Hospital), Ningbo Key Laboratory for Physical Diagnosis and Treatment of Mental and Psychological Disorders, Ningbo, Zhejiang 315201 China; 2 The Second Affiliated Hospital of Xinxiang Medical University, Xinxiang, Henan 453002 China; 3 Zhejiang Provincial Key Laboratory of Pathophysiology, Health Science Center, Ningbo University, Ningbo, Zhejiang 315211 China; 4 Department of Psychiatry, Yuyao Third People’s Hospital, Ningbo, Zhejiang 315599 China

**Keywords:** Depression, Physiology, Diagnostic markers

## Abstract

Major depressive disorder (MDD) with suicidal ideation (SI) significantly impacts global health. Suicidal ideation is associated with alterations in brain network connectivity, yet the effective connectivity from the dorsolateral prefrontal cortex (DLPFC) to functional network nodes remains poorly understood. This study utilizes transcranial magnetic stimulation-electroencephalography (TMS-EEG) to investigate DLPFC connectivity and cortical excitability changes, providing insights into the neurobiological mechanisms and potential treatments for MDD with SI. This study recruited 166 patients with MDD and 61 healthy controls. The TMS-EEG technique was used to assess effective connectivity based on abnormal time-locked TMS evoked potentials (TEPs). Suicidal ideation was assessed using the suicidality module of the Mini International Neuropsychiatric Interview (MINI), and participants were classified into suicidal ideation (SI) and non-SI (NSI) groups based on the presence of active ideation. Subgroup analysis evaluated significant current scattering (SCS) in DLPFC-related circuits through source localization, with multiple functional networks defined as downstream regions of interest. TEP analysis at the F3 electrode revealed no significant differences between the MDD and HC groups across components. However, the SI group exhibited increased N100 amplitudes compared to the NSI group (uncorrected) and healthy controls. Source-level brain network analysis showed that the SCS of the DLPFC-mPFC and DLPFC-ACC circuits was significantly greater in the SI group than in the NSI and control groups. After controlling for age, logistic regression analysis indicated a significant association between these connectivity patterns and the presence of suicidal ideation. MDD patients with suicidal ideation exhibit altered cortical inhibition and enhanced effective connectivity between the DLPFC and key brain regions, such as the ACC and mPFC. These exploratory findings contribute to a deeper understanding of the neural circuitry involved in suicidal ideation.

## Introduction

Major depressive disorder (MDD) is a prevalent and debilitating mental health condition that affects millions of individuals globally [1]. It is characterized by persistent and pervasive feelings of sadness, hopelessness, anhedonia, and an elevated risk of suicide [2]. Suicide, encompassing ideation, planning, and attempts, is a significant global public health issue, claiming over 800,000 lives annually [3]. Among U.S. adolescents, 4.1% to 8.6% (equivalent to 1.7–3.6 million individuals) have reported attempting suicide [4]. Suicidal ideation (SI) refers to the thoughts or plans of ending one’s own life, and it is a major risk factor for suicide attempts and death [5]. Revealing the neural basis of suicidal ideation can enhance our understanding of the neurobiological mechanisms underlying suicide, provide biomarkers for monitoring the progression of suicidal thoughts, assist in identifying individuals at risk, and facilitate the development of novel and effective interventions.

The factors underlying suicidal ideation are multifaceted and complex, involving disruptions in brain networks related to emotional regulation, impulse control, and decision-making [6]. Imbalances in excitatory and inhibitory neurotransmitter systems may also play a significant role in the emergence of suicidal thoughts. Previous studies have identified key neural networks, such as the default mode network (DMN), executive control network (ECN), and salience network (SN), that are associated with suicidal ideation [7, 8]. These networks are involved in emotional regulation, cognitive control, and self-referential thinking [9, 10], and their dysfunction is widely regarded as fundamental to psychiatric disorder [11], aligning with the triple-network model of psychopathology [12]. Resting-state fMRI studies have shown that reduced intrinsic connectivity in the left ECN, anterior DMN, and SN is associated with a higher severity of lifetime suicidal ideation [8]. Additionally, a magnetoencephalography (MEG) study [13] with source localization has found that the connectivity of inter-network gamma oscillations between the ECN and DMN is significantly negatively correlated with suicide risk, independent of depression severity. Furthermore, the DMN facilitates internally directed cognitive processes related to rumination [14] and may play a role in regulating the connection between intrinsic motivation, cognition, and control. For example, dysfunction in the DMN may lead to suicidal ideation through abnormal processing of psychological pain [15]. Compared with MDD without suicidal ideation, MDD with suicidal ideation had reduced functional connectivity within the ECN and DMN, and the strength of ECN connectivity was positively correlated with attention and alertness [16]. These studies have focused on the intrinsic functional connectivity of brain networks during rest, which reveals how regions collaborate, but they do not address the causal role one region plays in driving the activity of another. In other words, few studies have explored the effective connectivity of the brain under specific activation patterns. Effective connectivity refers to the causal relationships between brain regions—specifically how one region directly influences the activity of another. The dorsolateral prefrontal cortex (DLPFC) plays a critical role in top-down emotion regulation, cognitive control, and decision-making [17, 18], and the neural circuits associated with it may play a key role in modulating suicidal ideation. A recent meta-analysis combining data from 20 studies supports the DLPFC as a promising target for neuromodulation to reduce SI in individuals with depression [19]. Evidence suggests that the prefrontal cortex is involved in decision-making [20], impulse control [21], and emotional response inhibition [22], which are commonly associated with suicidal risk. However, the DLPFC, as a key regulatory center within the prefrontal system, has not been fully explored in terms of its top-down effective connectivity with critical nodes of brain networks in individuals with suicidal ideation.

The aim of this study was to investigate the effective connectivity patterns from DLPFC to functional network nodes in patients with MDD combined with SI using transcranial magnetic stimulation concurrent with electroencephalography (TMS-EEG) technology [23]. Compared to single-modal EEG or functional magnetic resonance imaging, TMS-EEG allows for the stimulation of neural circuits and the recording of their responses, enabling causal assessment [24]. By applying TMS to a target brain region and measuring EEG responses in other brain regions, TMS-EEG can directly assess neuronal activity, connectivity, and plasticity in the human brain, revealing trans-synaptic transmission and network modulation in the target brain region [25]. It enables us to measure the neural responses elicited by TMS stimulation of target brain regions, recording causal signals between brain networks with millisecond temporal resolution [26]. When a single-pulse TMS is applied to the cortical surface, it generates what are known as TMS-evoked potentials (TEPs) in the EEG [27]. TEPs are voltage changes that occur between electrodes within several hundred milliseconds following the TMS pulse, reflecting a series of alternating excitatory and inhibitory states in the cortical brain. For example, the classic TEPs induced by stimulation of the DLPFC are defined by the components P30, N45, P60, N100, and P180 [28]. TMS-EEG not only reveals changes in local cortical excitation and inhibition circuits but also provides deep insights into alterations in connectivity patterns [29, 30], offering a unique window to explore causal connectivity under different brain activation states. A limitation of TMS-EEG technology is that TMS pulses can induce artifacts. However, despite these challenges, test-retest reliability studies using artifact suppression techniques have demonstrated high reproducibility [31, 32]. The combination of TMS-EEG technology with signal source localization and brain network connectivity algorithms allows for exploring neural transmission characteristics between targeted brain regions and others. In recent years, this approach has become increasingly valuable in psychiatric research [33, 34]. We hypothesized that MDD individuals with suicidal ideation have impaired cortical excitatory or inhibitory function as evidenced by abnormal wave amplitudes of TEPs, and furthermore, that during the time-locked window of abnormal TEPs, these individuals exhibit alterations in neural connectivity patterns related to the DLPFC.

## Material and methods

### Participants

This study enrolled 166 patients with major depressive disorder (MDD) from Ningbo Kangning Hospital. All patients were independently diagnosed by two experienced psychiatrists in accordance with the Structured Clinical Interview for the Diagnostic and Statistical Manual of Mental Disorders (DSM-V). The inclusion criteria were: (1) Age between 18 and 65 (2) Hamilton Depression Scale-24 score (HAMD-24) ≥20 points; (3) Written informed consent from the patients or their guardians. The exclusion criteria were: (1) Patients with other psychiatric disorders or severe neurological diseases; (2) Patients with severe somatic diseases, infectious diseases, or immune system diseases. (3) Presence of a history of substance abuse. (4) Contraindications to magnetic fields, such as metallic implants in the head. We also recruited 61 demographically matched healthy controls (HC) in the local community, excluding individuals with anxiety and depressive symptoms, other major medical conditions, and brain injury.

**Fig. 1:**
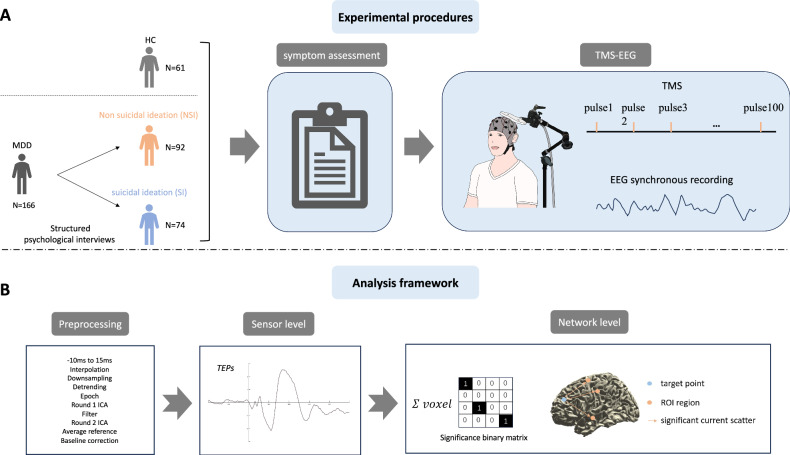
Overview of experimental design and data analysis. **A** Schematic diagram of the experimental procedure. All participants underwent clinical assessment (anxiety, depression) and TMS-EEG testing. After data collection was completed, patients were categorised into two subgroups with or without suicidal ideation based on interview. *HC*, health controls; *NSI*, Major depressive disorder with no suicidal ideation; *SI*, Major depressive disorder with suicidal ideation. **B** Process framework for TMS-EEG data analysis.

All participants received TMS-EEG testing and clinical assessment after enrollment (see Fig. 1A). Participants were assessed for symptoms of depression using the Hamilton Depression Scales (HAMD). The HAMD consists of 24 items that measure different aspects of depression, such as mood, anxiety, sleep, appetite, guilt, and suicidal ideation. Each item is rated on a scale of 0 to 4, except for a few items that are rated on a scale of 0 to 2. The total score ranges from 0 to 76, with higher scores indicating more severe depression. Two licensed neuropsychologists underwent training on the HAMD scale provided by professional assessment staff prior to the formal study. Two licensed neuropsychologists underwent training on the HAMD scale provided by professional assessment staff prior to the formal study. To ensure the reliability of the evaluations, the intraclass correlation coefficient (ICC) between the two neuropsychologists was required to be no less than 0.8. If the ICC fell below 0.8, discrepancies in the assessment process were resolved through consensus, followed by a new round of evaluations with two additional patients. Based on the structured diagnostic criteria of the Mini-International Neuropsychiatric Interview (MINI), participants with MDD were categorized into two groups: the SI group (MDD with suicidal ideation, MINI Suicide Module score greater than or equal to 6) and the NSI group (MDD without suicidal ideation). Obtaining information on patients’ antidepressant use and medical history from the hospital’s electronic records. All participants provided written informed consent in accordance with procedures approved by the Ethics Committee of Ningbo Kangning Hospital. Some participants in this study were also enrolled in a clinical trial registered in the Chinese Clinical Trial Registry (ChiCTR2100052122) at http://www.chictr.org.cn. This study was designed as an independent cross-sectional analysis conducted before the initiation of the interventional trial, with 67 participants diagnosed with MDD subsequently included in the trial.

### TMS-EEG testing and data preprocessing

Three experimenters conducted the TMS-EEG assessments for all participants, remaining blinded to the clinical evaluation information to ensure unbiased data collection and analysis. Three-dimensional T1-weighted images were collected using the uMR 890 3.0 T magnetic resonance imaging system (Lianying Co Ltd., Shanghai, China). These T1-weighted images were subsequently imported into the Brainsight TMS navigation system (Rogue Research Inc., Montreal, Canada) to guide the positioning of the stimulation coil. The stimulation site selected in this study corresponds to the average MNI coordinates reported in the literature within the DLPFC, which exhibit the strongest functional connectivity with other brain regions. Specifically, the MNI coordinates of the left DLPFC target stimulated in this study were [−44, 40, 29] [35, 36].

The TMS-EEG study was conducted using a Magstim figure-of-eight coil (D70-air film coil, Magstim Ltd, Oxford, UK) and a 64-channel EEG cap equipped with TMS-compatible Ag/AgCl electrodes (BrainProducts, Germany). The coil was tangential to the scalp with the handle pointing backwards at a 45° angle. Prior to recording TMS-EEG, the left primary motor cortex (M1) was stimulated to determine the individual’s resting motor threshold (RMT). The RMT was defined as the lowest stimulus intensity that produced a motor evoked potential (MEP) of at least 50 μV in the relaxed right adductor pollicis muscle in at least 5 out of 10 consecutive trials. The stimulation intensity was set at 100% RMT for all participants, and a total of 100 single-pulse TMS stimuli were delivered over the left DLPFC while the participants were instructed to relax and keep their eyes open. The time interval between pulses was fixed at 5 s with a random jitter of ±0.1–0.2 s to minimize anticipatory effects. We used 100% RMT to ensure participant safety and minimize discomfort, especially considering the inclusion of individuals with suicidal ideation. Pilot testing confirmed that this intensity reliably elicited motor-evoked potentials while maintaining comfort and compliance among participants. FCz and AFz are used as reference and ground electrodes, respectively. The signals were digitized at a high sampling rate of 25 kHz and recorded using BrainVision Recorder software (BrainProducts, Germany) to avoid amplifier saturation and minimize TMS-related artifacts. The electrode impedances were monitored throughout the experiment and maintained below 5 k Ω. To decrease the auditory evoked potentials induced by TMS stimulation, participants wore earplugs to block out noise during data acquisition.

We processed the recorded TMS-EEG data using a comprehensive data analysis framework (see Fig. 1B). Data were preprocessed using Matlab R2016b (MathWorks, USA) in combined with the EEGLAB [37] and ARTIST [38] toolboxes. The preprocessing code was adapted from a fully automated artifact suppression algorithm specifically designed for single-pulse TMS-EEG data. First, the EEG data within the time window containing the TMS pulse artifacts (from −10 to 25 ms) were removed and replaced with interpolated values to mitigate any sharp artifacts caused by the TMS pulse. Next, the EEG data were downsampled to 1 kHz to reduce file size and facilitate processing. The data were then segmented into epochs from 2 s before the stimulus to 2 s after the stimulus. After segmentation, the first round of independent component analysis (ICA) was applied to identify and remove large artifacts. Specifically, the *classifydecayart* function from the ARTIST toolbox was used to detect and remove decay components associated with TMS-induced artifacts, particularly those with high amplitudes and slow decays. Following the artifact removal, bandpass filtering (1–100 Hz) was applied to attenuate slow drifts and high-frequency noise, and notch filtering (48–52 Hz) was used to eliminate 50 Hz AC line noise artifacts. Bad trials with signal amplitudes exceeding 3 standard deviations from the test mean were then identified and removed. In addition, bad channels were identified and interpolated through their neighboring channels. To remove remaining artifacts, such as scalp muscle artifacts and eye artifacts, a second ICA was performed. Finally, the clean data were re-referenced using the averaging reference method, and baseline correction was applied using the average amplitude of the −550 ms to −50 ms time window.

### Sensor level analysis

We analyzed the TMS-evoked potentials (TEPs) of the F3 electrode (corresponding to left DLPFC) to investigate a series of excitatory and inhibitory electrophysiological activities induced by stimulation. All available trial data were used for stacked averaging to obtain time-locked phase-locked TEP components. The typical TEPs induced by stimulation of the DLPFC are usually defined as P30 (25–35 ms), N45 (40–50 ms), P60 (50–70 ms), N100 (80–120 ms), and P180 (160–200 ms).

### Neural network analysis

We performed source localization of the TMS-EEG data using the FieldTrip [39] toolbox and custom scripts to project the scalp EEG signals to the cortical source space. A standard boundary element model (BEM) was used for head modeling, based on the template head model provided by FieldTrip. This approach ensured a realistic volume conduction model for source reconstruction. Electrode positions were aligned to the head model using the standard 10–20 electrode system. Alignment included projecting the electrodes onto the scalp surface and interactive manual adjustments to ensure proper fit. After alignment, the electrode positions were used to compute the forward model and lead field matrix. A source grid with 8-mm spacing was constructed, resulting in 7452 grid points within the brain volume, covering both superficial and deep cortical regions. The noise covariance matrix was estimated using the pre-stimulus time window of individual trials (−500-0 ms). A linear constrained minimum variance (LCMV) beamformer is applied to estimate the activation in the source space. Generalized LCMV filters (−500–500 ms) were computed for pre- and post-stimulation to project the sensor signals into the source space in a certain functional relationship. The two-dimensional matrixes (voxel, time) characterizing brain activation for further analysis. To characterize the neurotransmission induced by activation, we used a significant current scattering (SCS) algorithm to calculate the effective connectivity between the target site and each of the important nodes of the brain networks [40]. SCS is a standardized measure for assessing activation propagation that measures the effective connectivity between source spatial voxels and stimulation sites. It is calculated as ${SCS}={ss}\left(x,t\right)\times d\left(x-{x}_{{st}im}\right)$, where the source strength $({ss})$ at each voxel $x$ and time point $t$ was determined by a binary matrix of significant sources across the whole brain. The significance of the sources was assessed by random permutation tests of the pre- and post-stimulus periods, using 1000 permuted data sets with a significance level of alpha = 0.05. This procedure controls the multiple comparison problem and identifies signal sources that show reliable changes in activity as a result of TMS stimulation. The $d\left(x-{x}_{{st}im}\right)$ is calculated as the distance of each source voxel from the stimulation site.

We selected three functional brain networks of interest that play an important role in the pathological mechanisms of suicidal ideation: Default Mode Network (DMN), Executive Control Network (ECN), and Salience Network (SN). Here we calculated the SCS of important nodes in these brain networks based on the AAL template [41], DMN: mPFC (labeled as ‘Frontal_Sup_Medial_L’, ‘Frontal_Sup_Medial_R’), PCC (‘Cingulum_Post_L’, ‘Cingulum_Post_R’), angular (‘Angular_L’, ‘Angular_R’); ECN: DLPFC (‘Frontal_Mid_L’, ‘Frontal_Mid_R’), PPC (‘Parietal_Inf_L’, ‘Parietal_Inf_R’); SN: ACC (‘Cingulum_Ant_L’, ‘Cingulum_Ant_R’), insula (‘Insula_L’, ‘Insula_R’), amygdala (‘Amygdala_L’, ‘Amygdala_R’). These regions have been consistently identified in the literature as critical hubs within their respective networks, contributing to the understanding of suicidal ideation and related pathological mechanisms.

### Statistical analysis

All statistical analyses were performed using the Statistical Package for the Social Sciences (SPSS 23, IBM) and Python. Differences in continuous variables between two groups were assessed using independent samples t-tests. For comparisons involving more than two groups, analysis of variance (ANOVA) was conducted, with post hoc corrections applied for multiple comparisons. When the assumption of normality was violated, the Wilcoxon signed-rank test was utilized. In cases where homogeneity of variances was not met, Welch’s ANOVA was employed to ensure the robustness of the results. Linear mixed-effects models were implemented to control for potential confounding effects of various clinical variables. All statistical tests were two-tailed, and *p-*values less than or equal to 0.05 were considered statistically significant.

## Results

### Demographic and clinical assessments

There were no significant differences between the MDD and HC groups in terms of age (M[SD], HC, 30.1[8.6]; MDD, 30.0[15.0]; *t* = 0.04, *p* = 0.97), gender (*χ²* = 0.005, *p* = 0.94) and education (M[SD], HC, 12.5[1.2]; MDD, 12.0[3.1]; *t* = 1.88, *p* = 0.06). Depression (M[SD], HC, 4.8[2.1]; MDD, 25.1[5.1]; *t* = −42.22, *p* < 0.001***) level were significantly higher in the MDD group than in the HC group.

The interview results revealed that out of 166 individuals with MDD, 74 cases (45%) were accompanied by suicidal ideation. Demographic and symptom information for the group with and without suicidal ideation in MDD is presented in Table 1. There were no significant differences between the NSI (no suicidal ideation) and SI (suicidal ideation) samples in terms of gender (*χ²* = 0.14, *p* = 0.71), educational attainment (*t* = −0.99, *p* = 0.33), and depression (*t* = −1.23, *p* = 0.22) levels. There was a significant difference in age between the NSI and SI groups (*t* = 4.52, *p* < 0.001***).

**Table 1:** Demographic and clinical information of the individuals with MDD.

	NSI ^a^ (*n* = 92)	SI ^b^ (*n* = 74)	*t*/*χ*^*2*^	*p*
	*M(SD)*	*M(SD)*		
Gender			0.14	0.71
male	31	27		
female	61	47		
Age	34.3(16.4)	24.7(11.0)	4.52	<0.001***
Education(years)	11.8(3.2)	12.2(3.1)	−0.99	0.33
Antidepressants type			0.21	0.98
Escitalopram	60	48		
Fluoxetine	7	7		
Sertraline	16	12		
Venlafaxine	9	7		
Duration of MDD (months)	54.8(64.0)	48.5(66.8)	0.44	0.66
History of suicide	13	34		
HAMD ^c^	24.7(4.6)	25.6(5.7)	−1.23	0.22

*NSI* ^*a*^, Major depressive disorder with no suicidal ideation; *SI* ^*b*^, Major depressive disorder with suicidal ideation; *HAMD* ^*c*^, Hamilton Depression Scale.***, indicates a statistically significant difference in age between the SI group and the NSI group.

### Sensor level

**Fig. 2:**
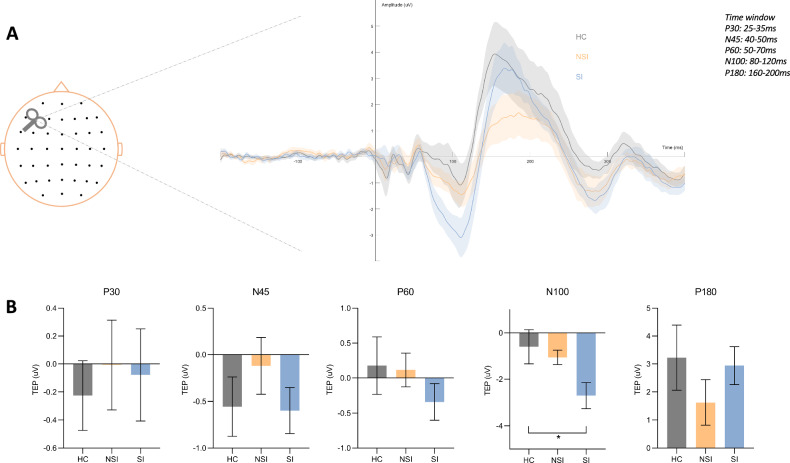
Sensor-level analysis of subgroups. **A** TMS evoked potentials (TEPs) recorded at the F3. **B** Comparison of the mean amplitude of TEPs between SI, NSI, and healthy controls groups.

To demonstrate the effectiveness of the preprocessing steps and the high quality of the data, group-averaged spectral analysis of the 2-second pre-TMS period and group-averaged TMS-EEG butterfly plot of preprocessed data are provided as Supplementary Materials. Quantification of TEP components at the F3 electrode was performed using an average amplitude algorithm to probe the electrophysiological activity induced by TMS pulses at the stimulation site, after re-referencing to the average (see Fig. 2A). The mean amplitude of each TEP component did not differ significantly between MDD and HC (for P30, *t* = −0.46, *p* = 0.65; for N45, *t* = −0.58, *p* = 0.56; for P60, *t* = 0.69, *p* = 0.49; for N100, *t* = 1.75, *p* = 0.08; for P180, *t* = 0.89, *p* = 0.38). We also compared TEP components between the HC, NSI and the SI groups. ANOVA results showed that the N100 component differed significantly among the three groups (*F*_*2, 224*_ = 4.32, *p* = 0.01*), N100 was significantly greater in the SI group than in the HC (*p* = 0.007**) and NSI groups (*p* = 0.02*). The post-hoc Tukey HSD test showed that, after correction, the N100 component difference between the HC and SI groups remained statistically significant (*p* = 0.02*), while the difference between the SI and NSI groups was not significant (*p* = 0.0516). No significant difference in P30 (*F*_*2, 224*_ = 0.12, *p* = 0.89), N45 (*F*_*2, 224*_ = 0.88, *p* = 0.42), P60 (*F*_*2, 224*_ = 0.88, *p* = 0.42), P180 (*F*_*2, 224*_ = 1.01, *p* = 0.37) components among the three groups (see Fig. 2B).

To evaluate whether N100 could serve as a potential biomarker for suicidal ideation, independent of other demographic and clinical factors such as age, duration of MDD symptoms, history of suicide attempts, and depression severity, we examined its ability to distinguish between SI and NSI groups. Linear mixed effects model was employed to control for the potential confounding effects of these variables on N100 amplitude. Group (SI vs. NSI), HAMD score, age, history of suicide attempts, and duration of MDD symptoms were included as fixed effects, with subject ID set as a random effect (see Table 2). The group variable (SI vs. NSI) was significantly associated with N100 amplitude (*β* = −1.447, *p* = 0.033*), the 95% confidence interval (CI) for the group coefficient was [−2.778, −0.116]. Duration of MDD symptoms was also significantly associated with N100 amplitude (*β* = 0.011, *p* = 0.036*), the 95% CI was [0.001, 0.021]. Both group (SI vs. NSI) and the duration of MDD symptoms significantly influence N100 amplitude. These findings suggest that suicidal ideation is associated with alterations in cortical inhibition, as reflected by the N100 amplitude. In contrast, other factors, including depressive symptom severity (HAMD), age, and history of suicide attempts, did not show significant associations with N100 in this study. Although electrode-level analyses are valuable for identifying time-locked neural activity, source-level significant current scattering (SCS) analysis is essential to confirm the cortical origins and functional network dynamics underlying these findings. The identified N100 time window will thus serve as the foundation for source-level analysis, providing deeper insights into the neural mechanisms associated with suicidal ideation.

**Table 2:** Linear mixed effects model for N100 and significant current scattering.

	Coef.	Std.Err.	Z	*P* Value	[0.025	0.975]
**N100**						
Intercept	−0.805	0.683	−1.179	0.239	−2.144	0.534
Group (SI vs. NSI)	−1.447	0.679	−2.131	**0.033**	−2.778	−0.116
HAMD	0.004	0.009	0.397	0.691	−0.014	0.022
Age	−0.022	0.024	−0.935	0.35	−0.069	0.025
History of suicide	−1.168	0.716	−1.631	0.103	−2.572	0.235
Duration of MDD	0.011	0.005	2.094	0.036	0.001	0.021
Group Var	7.584					
**SCS_mPFC** ^*a*^						
Intercept	832.435	141.437	5.886	0	555.224	1109.646
Group (SI vs. NSI)	216.843	99.121	2.188	**0.029**	22.569	411.117
HAMD	7.717	6.256	1.234	0.217	−4.544	19.979
Age	−7.354	3.727	−1.973	0.048	−14.658	−0.05
History of suicide	44.13	112.402	0.393	0.695	−176.173	264.434
Duration of MDD	0.078	0.807	0.096	0.923	−1.503	1.658
Group Var	188714.3					
**SCS_DLPFC** ^*b*^						
Intercept	1758.806	58.626	30	0	1643.901	1873.712
Group (SI vs. NSI)	297.308	175.885	1.69	0.091	−47.42	642.035
HAMD	11.063	7.289	1.518	0.129	−3.224	25.349
Age	−15.798	5.812	−2.718	0.007	−27.189	−4.407
History of suicide	42.618	184.09	0.232	0.817	−318.192	403.428
Duration of MDD	−0.403	1.345	−0.3	0.764	−3.039	2.232
Group Var	506074.4					
**SCS_ACC** ^*c*^						
Intercept	448.833	132.922	3.377	0.001	188.311	709.355
Group (SI vs. NSI)	108.946	42.373	2.571	**0.01**	25.896	191.996
HAMD	2.697	4.185	0.644	0.519	−5.506	10.9
Age	−3.651	1.275	−2.863	0.004	−6.15	−1.151
History of suicide	31.306	56.908	0.55	0.582	−80.233	142.844
Duration of MDD	−0.148	0.408	−0.363	0.716	−0.949	0.652
Group Var	46891.23					

*SCS_mPFC* ^*a*^, the significant current scatter from the stimulation target (F3/LDLPFC) to the medial prefrontal cortex; *SCS_DLPFC* ^*b*^, the significant current scatter from the stimulation target (F3/LDLPFC) to the dorsolateral prefrontal cortex; *SCS_ACC* ^*c*^, the significant current scatter from the stimulation target (F3/LDLPFC) to the anterior cingulate cortex. Bold *p*-values indicate statistically significant differences in SCS between SI and NSI groups after controlling for confounding variables.

### Neural network level

**Fig. 3:**
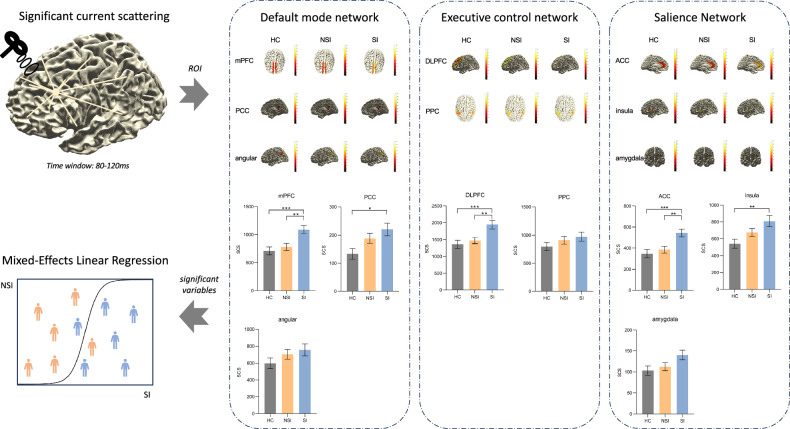
Comparison of SCS between stimulation target (F3/LDLPFC) and ROI nodes of each brain network, and then the inclusion of variables that differed significantly between the SI and NSI groups in the regression model for prediction of the presence or absence of suicidal ideation.

To identify the neural circuits that were associated with the abnormal GABAergic activity, we calculated the SCS of all ROIs in the time window that corresponded to the N100 component at the sensor level (see Fig. 3). In the Default Mode Network, mPFC varied significantly (*F*_*2, 224*_ = 8.08, *p* = 0.002**), with the SI group having a significantly higher SCS for mPFC than the NSI (*p* = 0.003**) and HC (*p* < 0.001***) groups. The SCS of PCC differed significantly among the three groups (*F*_*2, 224*_ = 4.35, *p* = 0.02*), SI group was significantly higher than that in the HC group (*p* = 0.01*). And the SCS of angular did not show significant differences among the three groups (*F*_*2, 224*_ = 1.43, *p* = 0.28). In the executive control network, DLPFC varied significantly (*F*_*2, 224*_ = 6.91, *p* = 0.003**), the SCS was significantly higher in the SI group than in the NSI (*p* = 0.003**) and HC (*p* < 0.001***) groups. The SCS of PPC did not show significant differences among the three groups (*F*_*2, 224*_ = 1.23, *p* = 0.30). In the Salience Network, the SCS of ACC (*F*_*2, 224*_ = 8.28, *p* = 0.002**), and insula (*F*_*2, 224*_ = 5.04, *p* = 0.01*) differed significantly among the three groups. In detail, the SCS of ACC was significantly higher in the SI group than in the NSI (*p* = 0.003**) and HC (*p* < 0.001***) groups. SCS in the insula (*p* = 0.005**) were significantly higher in the SI group than in the HC group. All p-values above have been corrected for appropriate multiple comparisons.

To verify whether the alterations were specific within the N100 time window, we performed additional statistical analyses of SCS across all component time windows in the Supplementary Materials, and the ANOVA results showed significant differences in SCS between the SI, NSI, and HC groups only during the 80–120 ms time window.

### Regression analysis

We examined whether network connectivity properties could distinguish between SI and NSI groups using Mixed Linear Model regression. The significant SCS values between the SI and NSI groups, along with demographic and clinical variables, were included as fixed effects in the regression model (see Table 2). We analyzed the connectivity between the stimulus target (F3/LDLPFC) and key brain network nodes—mPFC in the DMN, DLPFC in the ECN, and ACC in the SN—while controlling for age, depression symptoms, duration of MDD symptoms, and history of suicide. The results indicated that connectivity between the DLPFC and the mPFC (*p* = 0.029*) as well as the ACC (*p* = 0.01*) significantly distinguished the SI group from the NSI group. These findings highlight the importance of these network connections in predicting the presence of suicidal ideation in major depressive disorder (MDD), independently of other clinical factors.

## Discussion

This study revealed that (1) Although no significant differences in TEP component amplitudes were observed between MDD patients and healthy controls overall, those with suicidal ideation exhibited a greater N100 amplitude, suggesting altered cortical inhibitory function in the dorsolateral prefrontal cortex (DLPFC). (2) During the N100 time window, there is increased effective connectivity between the DLPFC and key regions such as the medial prefrontal cortex (mPFC) and anterior cingulate cortex (ACC) in MDD patients with suicidal ideation compared to those without, (3) and this alteration of connectivity has a predictive utility for suicidal ideation. In summary, we used the TMS-EEG technique to explore for the first time the neural mechanisms underlying abnormal directional connectivity between the DLPFC and brain functional networks during cortical inhibitory processes in MDD patients with suicidal ideation.

This study found that, compared to the HC and the NSI group, SI group exhibited significantly greater N100 amplitudes in the DLPFC. Although the difference in N100 amplitude between the SI and NSI groups did not survive multiple comparisons correction (*p* = 0.0516), this marginal effect may indicate that scalp-level measures alone are insufficiently sensitive to capture the subtle neural signatures of suicidal ideation. This aligns with accumulating evidence suggesting that suicidal ideation is associated with dysregulation across distributed functional networks, rather than localized cortical dysfunction [42–44]. Importantly, the robust difference between the SI and HC groups underscores the potential of the N100 component as a temporally specific neural marker of suicide-related pathophysiology, beyond general depressive symptoms. Moreover, our source-space effective connectivity analysis, constrained within the N100 time window, revealed significant group differences between SI and NSI, providing further support for the neurobiological relevance of this time window. These findings validate the use of the N100 as a functionally meaningful anchor point for probing network-level disturbances specifically linked to suicidal ideation. The N100 of TMS evoked potentials is a classical paradigm that is a reliable indicator for assessing the inhibitory properties of GABAergic interneurons [45–48]. The association between GABA-mediated cortical inhibitory dysfunction and suicidal ideation has been previously reported. For instance, cortical characteristics in depressed patients with severe suicidal ideation were assessed using the paired-pulse transcranial magnetic stimulation (ppTMS) paradigm [49]. The results demonstrated increased short-interval intracortical inhibition (SICI) and long-interval intracortical inhibition (LICI). Higher baseline SICI values were associated with more severe suicidal symptoms, and reductions in cortical inhibition following ketamine treatment, an NMDA receptor antagonist, were correlated with improvements in suicidal symptoms. Furthermore, cortical inhibition has been proposed as a potential biomarker for identifying depressed patients most likely to experience remission of suicidal ideation following magnetic seizure therapy (MST). TMS-EEG studies have demonstrated that baseline N100 amplitudes and LICI in the prefrontal cortex can serve as predictive indicators of suicidal ideation remission, with an accuracy of 89% [50]. Additionally, MST has been shown to enhance neuroplasticity in the prefrontal cortex through mechanisms akin to long-term potentiation (LTP), with reductions in cortical inhibition closely associated with the alleviation of suicidal ideation [51]. These findings align with the results of this study, underscoring the critical role of cortical inhibition in suicidal ideation and highlighting its potential as both a mechanistic marker and a therapeutic target. Notably, the absence of statistically significant TEP differences at the F3 electrode between MDD and HC participants in our study may seem inconsistent with findings from prior research [52], which reported increased N45, P60, and N100 amplitudes in MDD patients using the Global Mean Field Amplitude (GMFA) method. However, methodological differences could explain this apparent discrepancy. First, the GMFA reflects the overall strength of cortical responses by aggregating activity across all electrodes, thereby capturing more spatially distributed neural dynamics. In contrast, our study focused on TEPs recorded specifically at the F3 electrode, which primarily reflects localized activity within the left dorsolateral prefrontal cortex (lDLPFC). As such, GMFA may be more sensitive to subtle or spatially diffuse alterations in cortical excitability that are not readily observable at a single electrode site. Second, it is important to note that our study found a marginally significant difference in the N100 component (*p* = 0.08), which suggests a potential trend toward greater N100 in MDD, consistent with prior reports. While this effect did not reach the conventional threshold for significance, it may reflect a localized effect that is weaker or more variable compared to the global pattern captured by GMFA.

The enhanced connectivity between the DLPFC and mPFC/ACC in MDD patients with suicidal ideation reflects a potential compensatory mechanism for heightened emotional regulation demands and conflict monitoring. However, this overactivation might exacerbate self-referential negative processing and rumination, contributing to the persistence of suicidal thoughts. Existing research strongly supports the central role of the DLPFC in emotional regulation, cognitive control, and impulse inhibition [17], as well as the critical involvement of the ACC and mPFC in the evaluation and expression of negative emotions [53]. The enhanced connectivity between the DLPFC and the mPFC/ACC in MDD patients with suicidal ideation may reflect excessive mobilization of cognitive control mechanisms and heightened monitoring of emotional conflicts when confronted with intense negative emotions and stress. Evidence suggests that the ACC and mPFC may mediate the interaction between DLPFC activation and amygdala activation, as the prefrontal regions are implicated in reappraisal processes [54], whereas the lateral prefrontal cortex has minimal direct connections with the amygdala. Controlled top-down regulation, such as the modulation of emotional conflicts, relies on the ACC and mPFC to suppress negative emotion processing in the amygdala. Consequently, when suppression of limbic reactivity is required, the ventral ACC and mPFC may execute a generalized inhibitory function for negative emotions, which can be recruited by other regions, such as the DLPFC [53, 55]. In individuals with suicidal ideation, prolonged emotional stress and repetitive negative thinking may lead to an abnormal excitatory-inhibitory imbalance between the DLPFC and downstream emotion-related regions. DLPFC-associated circuits are linked to suicidal ideation and are important targets for some neuromodulatory treatments, such as transcranial magnetic stimulation [56, 57], and ketamine infusion [58, 59], that aim to restore the normal GABAergic functioning of the DLPFC and enhance the cognitive processes that impaired in suicidal patients. Suicidal ideation may be mediated by neural circuits involving DLPFC-mPFC, and DLPFC-ACC, which are responsible for selecting appropriate responses and inhibiting inappropriate ones [60–62]. These brain regions are involved in the regulation of stress and negative emotions, processes that are closely associated with suicide. Structural and functional abnormalities in the DLPFC, mPFC, and ACC have been consistently identified in patients with suicidal ideation, indicating disruptions in the cognitive and affective functions mediated by these regions. Notably, previous investigations have revealed reductions in gray matter volume, increased neuroinflammation, and decreased neural activity within the DLPFC and ACC of individuals with suicidality, compared to healthy controls or non-suicidal patients [63, 64]. These alterations likely reflect deficits in cognitive control, emotional regulation, and decision-making, which are critical for the adaptive modulation of distress and negative affect. The balance between excitatory and inhibitory processes within the mPFC is recognized as an important factor influencing mood and behavioral regulation [65]. Dysregulation in this balance could predispose individuals to heightened emotional reactivity and impaired stress coping mechanisms, thereby contributing to the development and persistence of suicidal ideation. Additionally, ACC interacts with the prefrontal and limbic regions [66] to appraise the negative affective valence and self-relevance of the stimuli and to regulate the emotional and behavioral responses accordingly [67–69], which is highly correlated with suicidal ideation and behavior [70]. These abnormalities may lead to a lack of effective strategies and control in coping with stress and negative emotions in patients with suicidal ideation, thus increasing the risk of suicide. This finding also holds significant clinical implications. The altered effective connectivity within DLPFC-related circuits may serve as a biomarker for predicting suicidal ideation, providing a novel diagnostic tool for early intervention. Furthermore, neuromodulation strategies targeting these brain regions could offer new avenues for treating suicidal ideation. Specifically, improving the neural circuits between the DLPFC-mPFC and DLPFC-ACC may be a critical therapeutic strategy for addressing suicidal thoughts.

This study has several limitations that should be acknowledged. First, it was an exploratory cross-sectional investigation, and evaluating cortical circuits using EEG is inherently challenging, particularly for deep brain targets such as the ACC. As a result, the role of these neural pathways in suicidal ideation requires further confirmation through future studies, including longitudinal and intervention-based research. Second, suicidal ideation in this study was assessed qualitatively, without quantifying the severity of suicidal thoughts. This limitation precluded the exploration of potential correlations between the severity of suicidal ideation and the observed electrophysiological indicators. Third, somatosensory-evoked potentials and the lack of control conditions, such as sham TMS or stimulation at a different site, may have influenced the observed results. Although consistent procedures were applied across participants to minimize confounding effects, the absence of a control condition remains a limitation. While the F3 electrode is commonly used in the literature as an approximate representation of left dorsolateral prefrontal cortex (lDLPFC) activity, we acknowledge that it does not precisely correspond to the exact MNI target coordinates used for TMS stimulation. The use of F3 was primarily intended to capture temporal characteristics of the cortical response, and not to delineate subregional specificity within the DLPFC. This limitation can be partially addressed by subsequent full electrode point-based source localization analyses. Lastly, the study utilized standard, rather than high-density, EEG data for source localization. These factors may have limited the precision of the connectivity analyses. Future studies could address these limitations by incorporating high-density EEG and individualized head models to achieve more accurate source localization. By acknowledging these limitations, we aim to provide a balanced interpretation of our findings and identify areas for improvement in future research.

## Conclusions

Using TMS-EEG technology, we identified abnormal cortical inhibition in the DLPFC of MDD patients with suicidal ideation, accompanied by heightened effective connectivity between the DLPFC and the ACC/mPFC. These findings provide novel insights into the neural mechanisms underlying suicidal ideation.

## Supplementary information

Supplementary Materials

## Data Availability

The data supporting the findings of this study are available from the corresponding author upon reasonable request.

## Code availability

Any code required to reproduce the results of this study can be obtained from the corresponding author upon request.
