# Supplementary material for: Heightened effective connectivity of DLPFC-mPFC and DLPFC-ACC circuits in major depressive disorder with suicidal ideation: evidence from a TMS-EEG study
Source: Transl Psychiatry. 2025 Aug 30;15:332. doi: 10.1038/s41398-025-03515-z (PMC12398619; doi:10.1038/s41398-025-03515-z)
Supplement: Supplementary file 1 — Supplementary Materials [file 41398_2025_3515_MOESM1_ESM.docx]

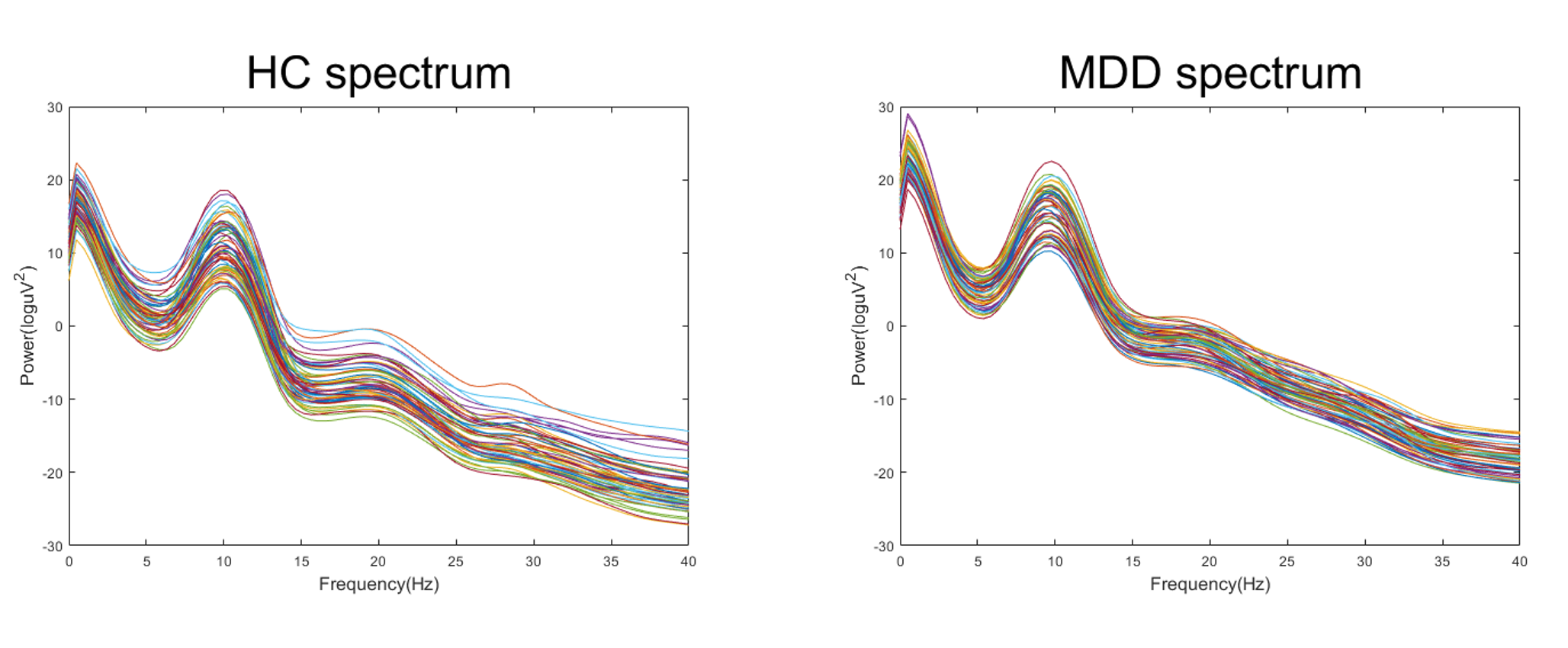


**Figure S1.** Spectral Analysis of Pre-TMS Data (2 seconds prior to TMS).


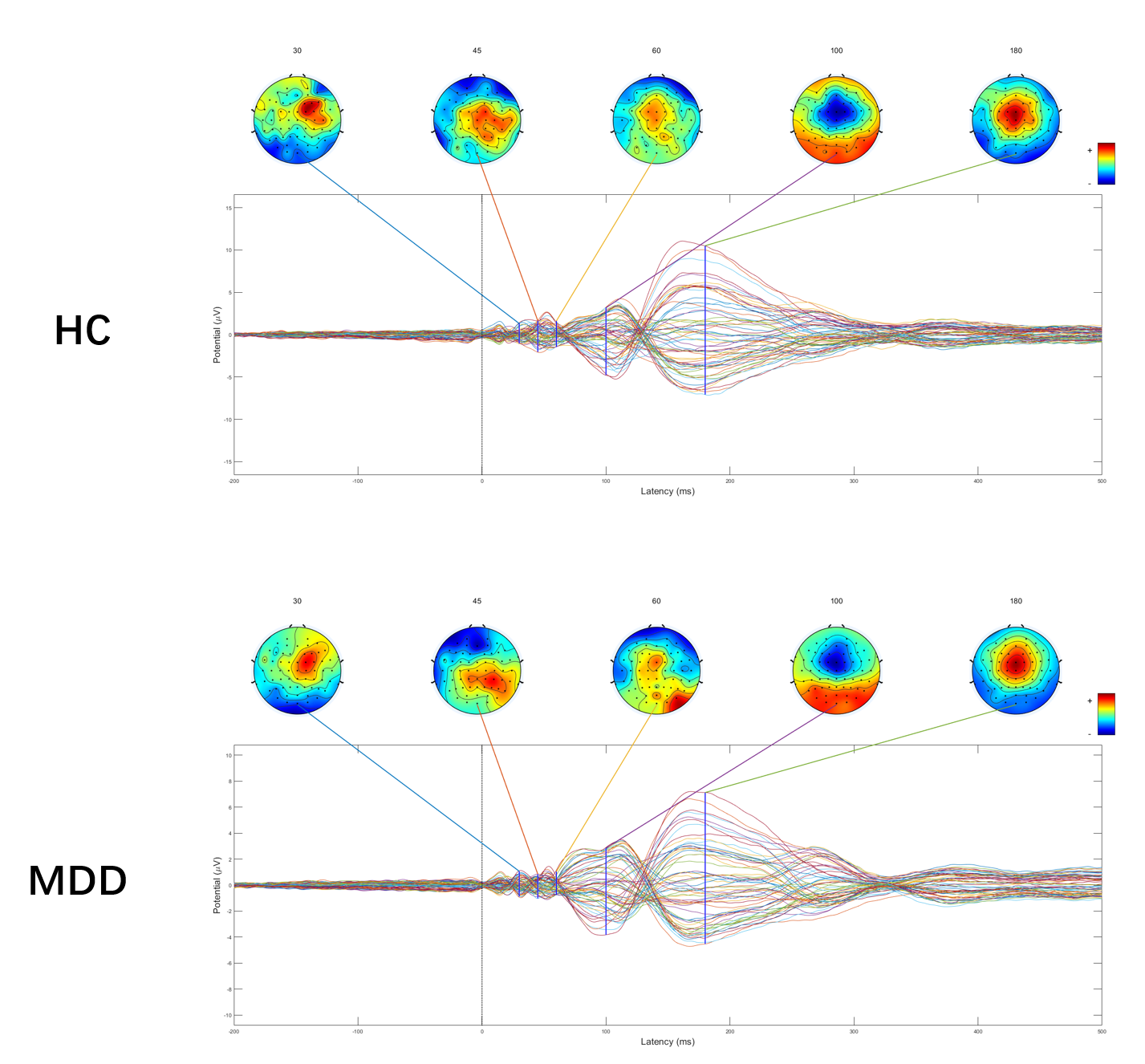


**Figure S2.** TMS-EEG Butterfly Plot of Preprocessed Data.


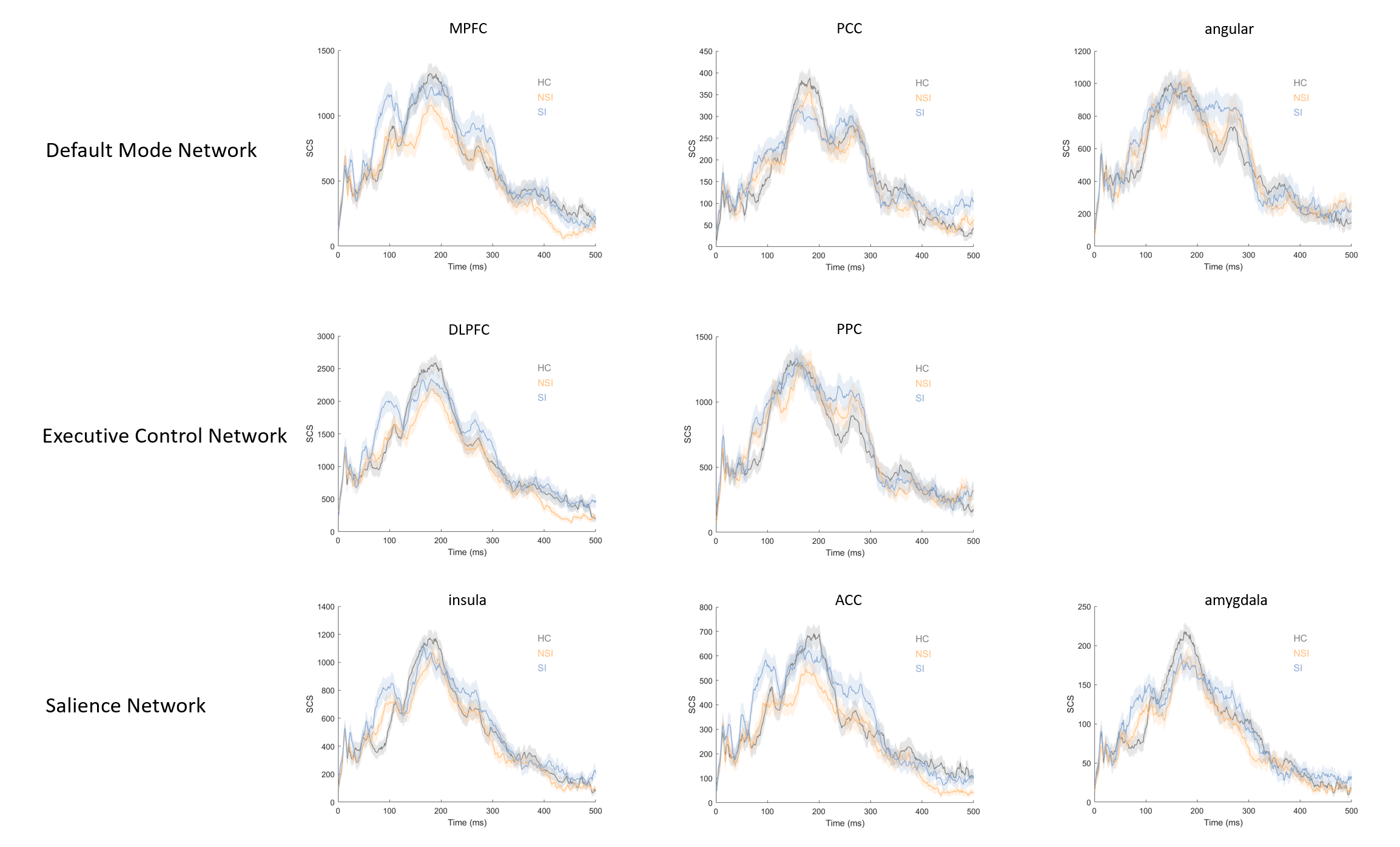


**Figure S3.** The trend of SCS over time (0-500ms) for each node of the brain networks.

**Table S1.** Analysis of variance (*p*-value) between the three groups within each component time window for each ROI node SCS of the brain network.

|  | P30(25-35ms) | N45(40-50ms) | P60(50-70ms) | N100(80-120ms) | P180(160-200ms) |
| --- | --- | --- | --- | --- | --- |
| MPFC | 0.408 | 0.747 | 0.320 | <0.001**^***^** | 0.084 |
| PCC | 0.890 | 0.437 | 0.603 | 0.014**^*^** | 0.325 |
| Angular | 0.893 | 0.287 | 0.728 | 0.242 | 0.837 |
| DLPFC | 0.686 | 0.446 | 0.310 | 0.001**^**^** | 0.072 |
| PPC | 0.950 | 0.430 | 0.629 | 0.295 | 0.953 |
| Insula | 0.599 | 0.790 | 0.437 | 0.007**^**^** | 0.314 |
| ACC | 0.395 | 0.583 | 0.244 | <0.001**^***^** | 0.057 |
| Amygdala | 0.689 | 0.423 | 0.413 | 0.049**^*^** | 0.088 |

**Table S2.** FDR-corrected *p*-values for the results in Table S1.

|  | P30(25-35ms) | N45(40-50ms) | P60(50-70ms) | N100(80-120ms) | P180(160-200ms) |
| --- | --- | --- | --- | --- | --- |
| MPFC | 0.519 | 0.558 | 0.478 | 0.005**^**^** | 0.236 |
| PCC | 0.611 | 0.444 | 0.528 | 0.071 | 0.459 |
| Angular | 0.597 | 0.561 | 0.560 | 0.559 | 0.591 |
| DLPFC | 0.562 | 0.436 | 0.525 | 0.010**^*^** | 0.229 |
| PPC | 0.619 | 0.475 | 0.532 | 0.535 | 0.605 |
| Insula | 0.544 | 0.573 | 0.463 | 0.046**^*^** | 0.499 |
| ACC | 0.528 | 0.549 | 0.518 | 0.009**^**^** | 0.207 |
| Amygdala | 0.547 | 0.488 | 0.500 | 0.207 | 0.222 |
